# Effects of blood flow restriction combined with low-load resistance training on obstacle-crossing performance and lower limb function in older adults

**DOI:** 10.3389/fspor.2026.1858963

**Published:** 2026-07-01

**Authors:** Zhengbin Li, Yang Liu, Fei Gao, Na Liu

**Affiliations:** College of Human Movement Science, Jilin Sport University, Changchun, China

**Keywords:** balance performance, blood flow restriction, low-load resistance training, obstacle crossing, older adults, toe clearance

## Abstract

**Background:**

Obstacle crossing is a demanding locomotor task for older adults and may be particularly sensitive to training-related changes in lower-limb function and balance-related performance. This study compared the effects of blood flow restriction combined with low-load resistance training (BFR-LLRT) and low-load resistance training alone (LLRT) on obstacle-crossing performance, neuromuscular activation, and balance-related outcomes in older adults.

**Methods:**

Forty community-dwelling older adults aged 60 years or above were randomly assigned to an LLRT group or a BFR-LLRT group and completed an 8-week intervention. Before and after training, participants were assessed for lower-limb muscle activation using surface electromyography, balance performance using computerized dynamic posturography, and obstacle-crossing characteristics using three-dimensional motion capture.

**Results:**

Both groups showed changes or improvements in selected neuromuscular, obstacle-crossing, and balance-related outcomes after training. Compared with LLRT alone, BFR-LLRT was associated with a greater increase in ankle co-activation before heel strike, a greater increase in trailing-limb toe clearance, and a larger increase in crossing velocity. Leading-limb toe clearance increased significantly in both groups, but the group × time interaction did not reach statistical significance. In balance testing, BFR-LLRT was associated with greater improvements in selected mediolateral rhythmic weight-shift conditions. However, weight-bearing symmetry showed angle-dependent and mixed responses: asymmetry increased at 30° and 60° knee flexion in the BFR-LLRT group, indicating poorer symmetry at these angles, whereas symmetry improved at 90°.

**Conclusion:**

BFR-LLRT may provide additional benefits over LLRT alone for selected aspects of laboratory-based, task-specific lower-limb function in older adults, particularly during more demanding components of obstacle crossing and dynamic balance control.

## 1 Introduction

Falls remain a major public health concern in older adults, with approximately one-third of community-dwelling older individuals experiencing at least one fall annually (1, 2). Many falls occur during locomotor tasks requiring environmental negotiation, such as obstacle crossing, which imposes substantial demands on dynamic balance (3), lower-limb control (4), and accurate foot placement (5). Impaired obstacle-crossing performance has been associated with increased tripping risk (6), gait asymmetry (7) and reduced functional mobility (8) in aging populations.

Successful obstacle negotiation depends on the coordinated contribution of lower-limb strength (9), postural control (10), and task-specific neuromuscular regulation (11). Age-related declines in muscle function and balance capacity may reduce toe clearance, impair weight transfer, and compromise landing stability during obstacle crossing, thereby increasing the likelihood of instability and falls (5, 12, 13). In particular, safe loading of the leading limb at landing is critical for maintaining forward progression while minimizing loss of balance (6, 14).

Resistance training is widely recommended to improve lower-limb function and preserve mobility in older adults (15–18). However, conventional high-load resistance exercise may not be feasible for all older individuals because of joint discomfort, reduced exercise tolerance, or safety concerns (19). BFR combined with low-load resistance training has emerged as an alternative approach that may improve selected muscle-related and functional outcomes at relatively low mechanical loads (20, 21). Recent evidence has supported the effectiveness of BFR-based interventions in improving muscle strength, physical function, and mobility-related outcomes in older adults (22–25), and experimental studies have reported that BFR-based walking or resistance training may improve selected functional performance outcomes in this population (26–28).

From a theoretical perspective, BFR-LLRT may be relevant to obstacle negotiation because lower-limb muscle-related function is involved in key components of obstacle crossing, including limb advancement, foot clearance, controlled landing, and forward progression (5, 6, 9, 13). In addition, obstacle negotiation requires timely activation and co-activation of muscles around the knee and ankle to regulate joint position and respond to landing demands (29–32). Therefore, examining EMG-derived co-activation around heel strike may provide useful information about training-associated changes in antagonist–agonist activation patterns during this task. However, evidence for task-specific locomotor outcomes after BFR-based training, particularly obstacle crossing, remains limited, especially in studies incorporating biomechanically quantified obstacle-crossing tasks.

Despite this growing evidence, few studies have examined whether BFR-based training is associated with changes in complex locomotor tasks such as obstacle crossing. In addition to lower-limb strength, obstacle negotiation involves timely muscle activation around landing and coordinated antagonist–agonist activity around the lower-limb joints (32). Co-activation of agonist–antagonist muscle groups around the knee and ankle is associated with joint stabilization demands during demanding gait tasks (29–31). Because leading- and trailing-limb obstacle crossing require foot clearance, controlled limb advancement, and stable landing, changes in knee and ankle co-activation around heel strike may be functionally relevant to laboratory-based obstacle-crossing performance. Therefore, examining muscle activation patterns around heel strike may help explore whether BFR-LLRT is associated with additional effects beyond those achieved with LLRT alone.

Accordingly, the present study investigated the effects of blood flow restriction combined with low-load resistance training on obstacle-crossing performance and lower-limb function in older adults. Specifically, we examined obstacle-crossing kinematics, balance performance, and neuromuscular co-activation during heel strike. We hypothesized that BFR-LLRT would be associated with greater changes or improvements in selected outcomes than LLRT alone.

## 2 Materials and methods

### 2.1 Study design

This study employed a randomized, parallel-group, pretest-posttest design to compare the effects of LLRT and BFR-LLRT on obstacle-crossing performance and lower-limb function in older adults. Outcome assessments were conducted before and after an 8-week intervention period. The study protocol was approved by the Academic Ethics Committee of Jilin Sport University (Approval No. 2025006), and all participants provided written informed consent prior to participation.

Participants were randomly allocated to either the LLRT or BFR-LLRT group using a computer-generated randomization sequence created by an investigator who was not involved in outcome assessment. Group assignments were maintained by this investigator until completion of baseline assessments. Due to the nature of the exercise intervention, participant blinding was not possible. Outcome assessors were not involved in the randomization procedure. Data processing and statistical analyses were performed using standardized procedures. The data analyst was not blinded to group allocation.

*A priori* sample size estimation was performed using G*Power 3 for a two-way mixed-design ANOVA (group × time interaction) 33). The calculation was based on the primary outcome, trailing-limb toe clearance during obstacle crossing. Previous studies have shown that BFR-based exercise can improve lower-limb strength, mobility, and functional performance in older adults (21–28). Toe clearance during obstacle crossing has been recognized as an important kinematic indicator related to gait safety and tripping risk (5, 6, 13, 34, 35). However, no previous study has specifically examined the effects of BFR-LLRT on obstacle-crossing performance, particularly trailing-limb toe clearance, in older adults. Therefore, a conventional medium effect size (f = 0.25), as defined by Cohen for ANOVA-based power analysis, was used for the estimation (36). The parameters were set as α = 0.05, power = 0.80, a correlation among repeated measures of 0.50, and a nonsphericity correction of 1.00. The analysis indicated that a minimum total sample size of 34 participants was required. To account for possible attrition and to ensure adequate statistical power, we aimed to recruit more participants than the minimum required sample size. Participant screening, exclusion before randomization, allocation, follow-up, and analysis are shown in Supplementary Figure S1.

### 2.2 Participants

Forty community-dwelling older adults aged 60 years or above were recruited. Participants were eligible if they were able to walk independently without an assistive device and had no self-reported or clinically apparent neurological, musculoskeletal, visual, or auditory impairments that could substantially affect gait or balance. Exclusion criteria included severe cardiovascular or cerebrovascular disease, lower-limb injury or surgery within the previous 6 months, psychiatric disorders, cognitive impairment that prevented understanding or following test instructions, or the use of medications that might influence postural control. All participants were screened for contraindications to resistance exercise and blood flow restriction training prior to enrollment.

Participants were randomly assigned to either the LLRT group or the BFR-LLRT group, with 20 participants in each group. Baseline characteristics were compared between groups to confirm initial comparability.

### 2.3 Intervention protocols

Both groups completed supervised lower-limb resistance training three times per week for 8 consecutive weeks. Each session was supervised by trained research staff and included a standardized warm-up, three lower-limb resistance exercises, and a cool-down period. The exercises consisted of straight leg raises, standing calf raises, and seated knee extensions. Straight leg raises and seated knee extensions were performed with individualized external loads, whereas standing calf raises were performed using body weight only. The training loads are summarized in Supplementary Table S1. Although these exercises were not obstacle-specific, they were selected to target lower-limb muscle groups involved in limb advancement, knee extension control, ankle plantarflexion, forward progression, and postural support during walking and obstacle negotiation. Thus, the intervention was intended to improve basic lower-limb functional capacity that may support laboratory-based obstacle-crossing performance, rather than to provide task-specific obstacle-crossing practice.

For externally loaded exercises, training intensity was prescribed at approximately 30%–40% of estimated one-repetition maximum (1RM), consistent with recommendations for low-load resistance training in older adults and commonly used BFR exercise protocols (15, 20, 21). For safety reasons, 1RM for externally loaded exercises was estimated using a submaximal procedure rather than a direct maximal test. Before 1RM estimation, participants completed a familiarization session to learn the correct technique for each externally loaded exercise. During testing, participants first performed a standardized warm-up with a light load. The external load was then gradually increased until the participant could complete approximately 8–12 repetitions with correct technique and without pain, compensatory movement, or breath-holding. The test was terminated when the participant could no longer complete a repetition with correct form or when pain, dizziness, excessive fatigue, abnormal cardiovascular symptoms, numbness, paraesthesia, or discomfort was reported. At least 2–3 min of seated rest was provided between attempts. Estimated 1RM was calculated using the Epley equation [estimated 1RM = load × (1 + repetitions/30)], a commonly used submaximal prediction equation that has been evaluated in older adults (37).

For externally loaded exercises, if a participant completed the prescribed sets and repetitions with correct technique, no marked compensatory movement, and no excessive fatigue or discomfort, the load was increased by approximately 0.5–1 kg for the next session while remaining within the target range of 30%–40% estimated 1RM. If a participant reported excessive fatigue, pain, numbness, paraesthesia, or delayed-onset muscle soreness lasting more than 48 h, the external load was maintained or reduced at the next session. For standing calf raises, progression was controlled by movement quality, completion of repetitions, and participant tolerance because the exercise was performed using body weight only.

The LLRT group performed each exercise for 3 sets of 30 repetitions, with 30–60 s rest intervals between sets. The initial rest interval was 30 s, but it could be extended to 60 s when needed according to participant tolerance, recovery status, and safety monitoring. Rest intervals were extended if participants reported excessive fatigue, discomfort, numbness, paraesthesia, dizziness, or delayed recovery. This rest interval was selected to maintain the low-load, high-repetition training stimulus while allowing sufficient recovery and safety monitoring in older adults.

The BFR-LLRT group performed the same exercise program with the addition of blood flow restriction applied bilaterally to the proximal thighs using inflatable pneumatic cuffs/sleeves (B-STRONG, USA). Size #4 cuffs were used. According to the manufacturer's specifications, the cuffs were 7 cm in width and adjustable from 54 to 79 cm in length, corresponding to a recommended limb circumference range of 56.5–73.5 cm. The cuffs were positioned at the most proximal portion of each thigh. Cuff size and position were kept consistent for each participant across all training sessions.

BFR pressure was prescribed relative to each participant's arterial occlusion pressure (AOP), in accordance with current methodological recommendations for individualized BFR exercise (20, 38). AOP was determined using Doppler ultrasound, as described in Section 2.4. To improve tolerance and safety while maintaining an adequate BFR stimulus, the relative cuff pressure was progressively increased across the intervention period: 50% AOP during weeks 1–2, 60% AOP during weeks 3–5, and 70% AOP during weeks 6–8. Cuff pressure was maintained during each exercise set, released during the 30–60 s inter-set rest period, and reapplied before the subsequent set. This individualized, progressive, and intermittent AOP-based strategy was used to improve the consistency, safety, and tolerability of BFR application in older adults (20, 38).

Participants were screened for contraindications to resistance exercise and blood flow restriction training before enrollment. During every session, participants were verbally monitored for excessive fatigue, pain, numbness, paraesthesia, pressure discomfort, dizziness, chest discomfort, abnormal shortness of breath, and delayed-onset muscle soreness. Although formal RPE scores were not recorded using a standardized scale, perceived exertion and exercise tolerance were monitored through standardized verbal questioning and observation by trained research staff. Delayed-onset muscle soreness was checked verbally before the next training session. Training was stopped or modified if a participant reported marked pain, numbness, paraesthesia, dizziness, chest discomfort, abnormal shortness of breath, excessive fatigue, or delayed-onset muscle soreness lasting more than 48 h. All adverse events were recorded throughout the intervention. Training adherence was calculated as the percentage of supervised training sessions completed out of the 24 prescribed sessions. A session was considered completed when the participant attended the supervised session and completed all prescribed exercises, with exercise modification permitted when needed for safety. The actual training loads, AOP values, applied cuff pressures, adherence, and safety outcomes are summarized in Supplementary Table S1.

### 2.4 Arterial occlusion pressure assessment

AOP was determined using a portable Doppler ultrasound device (TE7, Mindray, China), following established BFR assessment procedures (20, 38). Participants rested quietly before testing. The posterior tibial artery was identified, and cuff pressure was gradually increased until arterial pulse cessation was detected. Two measurements were obtained, and a third measurement was performed if the difference between trials exceeded 15 mmHg. The average value was used to determine individualized BFR pressure during training. This approach is commonly used to individualize cuff pressure and support the safe application of BFR interventions.

### 2.5 Experimental procedures

Obstacle-crossing trials were performed barefoot under controlled laboratory conditions at a self-selected walking speed. Participants were instructed to “walk at your normal comfortable speed and step over the obstacle as naturally as possible without contacting it”. No instruction was given to walk faster or slower. The walkway length was 8 m, and the obstacle was positioned approximately at the midpoint of the walkway to allow participants to reach a stable approach speed before crossing.

Obstacle height was set at 10% of each participant's leg length, consistent with previous obstacle-negotiation research in older adults (5, 6, 13, 34). Leg length was measured from the anterior superior iliac spine to the medial malleolus. Participants were allowed several familiarization trials before data collection. Thereafter, three successful trials were recorded and averaged. A successful trial was defined as crossing the obstacle without foot-obstacle contact, obvious stumbling, stopping before the obstacle, use of external support, an additional recovery step immediately after crossing, or assistance from the examiner. Trials were repeated if any of these events occurred.

Obstacle crossing was performed using the naturally selected leading limb. The leading limb was not constrained to be the dominant or non-dominant limb, in order to preserve natural obstacle-crossing behavior under self-selected walking conditions.

### 2.6 Outcome measures

The primary outcome was trailing-limb toe clearance during obstacle crossing, because this variable reflects foot-obstacle clearance during the more demanding phase of obstacle negotiation and is directly related to tripping avoidance in older adults. Secondary outcomes included leading-limb toe clearance, crossing velocity, knee and ankle co-activation ratios around heel strike, Rhythmic Weight Shift on-axis velocity, and Weight-Bearing Squat symmetry.

#### 2.6.1 Surface electromyography

Surface electromyography (sEMG) signals were recorded using a wireless system (BTS FreeEMG, BTS S.p.A., Italy) at a sampling frequency of 1,000 Hz. Electrodes were placed on the rectus femoris, vastus medialis, vastus lateralis, biceps femoris, gastrocnemius, and tibialis anterior according to SENIAM recommendations (39). Muscle selection was based on their established roles in lower-limb control and obstacle-related balance recovery during walking (31, 35, 40, 41).

Raw EMG signals were band-pass filtered at 20–500 Hz, notch filtered at 50 Hz, and full-wave rectified. RMS values were calculated to quantify muscle activation amplitude. EMG amplitudes were normalized to the peak value obtained across all valid obstacle-crossing trials for each participant. Heel-strike events were identified from synchronized kinematic data, and RMS values within 50 ms before and after heel strike were extracted to represent pre-activation and post-activation.

Co-activation ratios were calculated to describe antagonist–agonist activation patterns around heel strike. For the knee joint, quadriceps activation was defined as the mean RMS of rectus femoris, vastus medialis, and vastus lateralis, and the co-activation ratio was calculated as biceps femoris activation divided by quadriceps activation. For the ankle joint, the co-activation ratio was calculated as tibialis anterior activation divided by gastrocnemius activation. These indices were used as EMG-derived measures of antagonist–agonist co-activation around the joint during obstacle crossing (31, 41).

#### 2.6.2 Balance performance

Balance performance was assessed using a computerized dynamic posturography system (NeuroCom Balance Manager, Natus Medical, USA), which has been widely used to evaluate postural control in older adults in both clinical and research settings (42–44).

In the Rhythmic Weight Shift (RWS) test, participants voluntarily shifted their center of gravity in the mediolateral and anteroposterior directions under slow, moderate, and fast conditions, as commonly implemented in computerized dynamic posturography assessments (43, 45). The primary variable analyzed was on-axis velocity, representing the speed of controlled weight shifting (46).

The Weight-Bearing Squat (WBS) test was used to assess lower-limb loading symmetry at knee flexion angles of 0°, 30°, 60°, and 90°. The percentage of body weight supported by the left and right limbs was recorded at each angle. Weight-bearing symmetry was quantified as the absolute deviation of unilateral weight-bearing percentage from 50%, a commonly used approach for evaluating bilateral loading asymmetry (47, 48), with smaller values indicating a more symmetrical distribution of body weight.

#### 2.6.3 Obstacle-crossing performance

Obstacle-crossing kinematics were recorded using a three-dimensional motion capture system (Codamotion, Charnwood Dynamics Ltd., UK) at a sampling frequency of 100 Hz. Reflective markers were placed on anatomical landmarks of the pelvis and lower limbs to reconstruct segment motion. Participants walked at a self-selected speed and stepped over the obstacle.

The obstacle-crossing kinematic variables included leading-limb toe clearance, trailing-limb toe clearance, and crossing velocity. Toe clearance was defined as the vertical distance between the toe and the obstacle at the moment of crossing, a key determinant of tripping risk during obstacle negotiation (5, 13, 34, 35).

Crossing velocity was calculated as the anteroposterior displacement of the midpoint of the bilateral anterior superior iliac spines from leading-limb toe-off to trailing-limb heel strike divided by the corresponding time interval, consistent with previous studies quantifying forward progression during obstacle crossing (6, 10, 34, 49).

### 2.7 Data processing

Kinematic data were low-pass filtered using a fourth-order Butterworth filter with a cutoff frequency of 6 Hz. EMG and kinematic data were synchronized before analysis. For each participant, three valid trials, as defined in the experimental procedures, were averaged to obtain representative values. The same processing pipeline was applied to both pre-intervention and post-intervention data, consistent with standard obstacle-crossing gait analyses reported in previous studies (34, 50).

### 2.8 Statistical analysis

All statistical analyses were conducted using R (version 4.5.3; R Foundation for Statistical Computing, Vienna, Austria). Data are presented as mean ± standard deviation or number of participants, as appropriate. Normality was assessed using the Shapiro–Wilk test. Baseline differences between groups were evaluated using independent-samples t tests for continuous variables. Categorical baseline variables, including sex distribution, dominant limb, diabetes, hypertension, and fall history in the past 12 months, were compared using Fisher's exact tests because of the small sample size. Fall history was not statistically compared because no participant in either group reported a fall in the past 12 months.

Intervention effects were analyzed using a two-way mixed-design ANOVA (group × time), with time (pre vs. post) as the within-subject factor and group (LLRT vs. BFR-LLRT) as the between-subject factor, across all outcome variables. The primary analysis focused on the group × time interaction for the primary outcome, trailing-limb toe clearance. Analyses of secondary outcomes were considered secondary and were interpreted in relation to the primary outcome. When significant group × time interactions were identified, Bonferroni-adjusted *post hoc* comparisons were performed. Statistical significance was set at *p* < 0.05 (two-tailed), and effect sizes were reported as partial *η*^2^.

## 3 Results

### 3.1 Participant characteristics

A total of 46 older adults were assessed for eligibility. Six individuals were excluded before randomization because they did not meet the eligibility criteria, declined to participate, were unable to attend the intervention regularly, or could not be contacted. Finally, 40 participants were randomized, with 20 allocated to the LLRT group and 20 allocated to the BFR-LLRT group. All randomized participants completed the intervention and were included in the final analysis.

No significant between-group differences were observed in the available baseline characteristics, including age, sex distribution, height, body mass, dominant limb, diabetes, or hypertension (Table 1). No participant in either group reported a fall in the past 12 months. Participants with diabetes or hypertension were medically stable, and their conditions were controlled with medication. All participants were able to walk independently without an assistive device. These findings suggest that the two groups were comparable based on the measured baseline variables.

**Table 1:** Baseline characteristics of the participants.

Variable	LLRT (*n* = 20)	BFR-LLRT (*n* = 20)	Mean difference (95% CI)	Test statistic	*p*
Age, years	66.89 ± 4.31	67.62 ± 3.74	−0.74 (−3.32, 1.84)	−0.580	0.565
Sex, male/female, n	8/12	9/11	—	Fisher's exact	1.000
Height, cm	161.57 ± 6.09	162.05 ± 6.61	−0.48 (−4.55, 3.59)	−0.239	0.812
Body mass, kg	62.08 ± 4.86	62.61 ± 3.53	−0.54 (−3.26, 2.19)	−0.398	0.693
Fall history in past 12 months, yes/no, n	0/20	0/20	—	Not tested	—
Dominant limb, right/left, n	19/1	20/0	—	Fisher's exact	1.000
Diabetes, yes/no, n	1/19	3/17	—	Fisher's exact	0.605
Hypertension, yes/no, n	11/9	8/12	—	Fisher's exact	0.527

Data are presented as mean ± SD or number of participants. Mean difference was calculated as LLRT minus BFR-LLRT. Continuous variables were compared using independent-samples t tests. Categorical variables were compared using Fisher's exact tests because of the small sample size. Fall history was not statistically compared because no participant in either group reported a fall in the past 12 months. Participants with diabetes or hypertension were medically stable, and their conditions were controlled with medication. LLRT,  low-load resistance training; BFR-LLRT, blood flow restriction combined with low-load resistance training.

### 3.2 Neuromuscular activation

**Figure 1:**
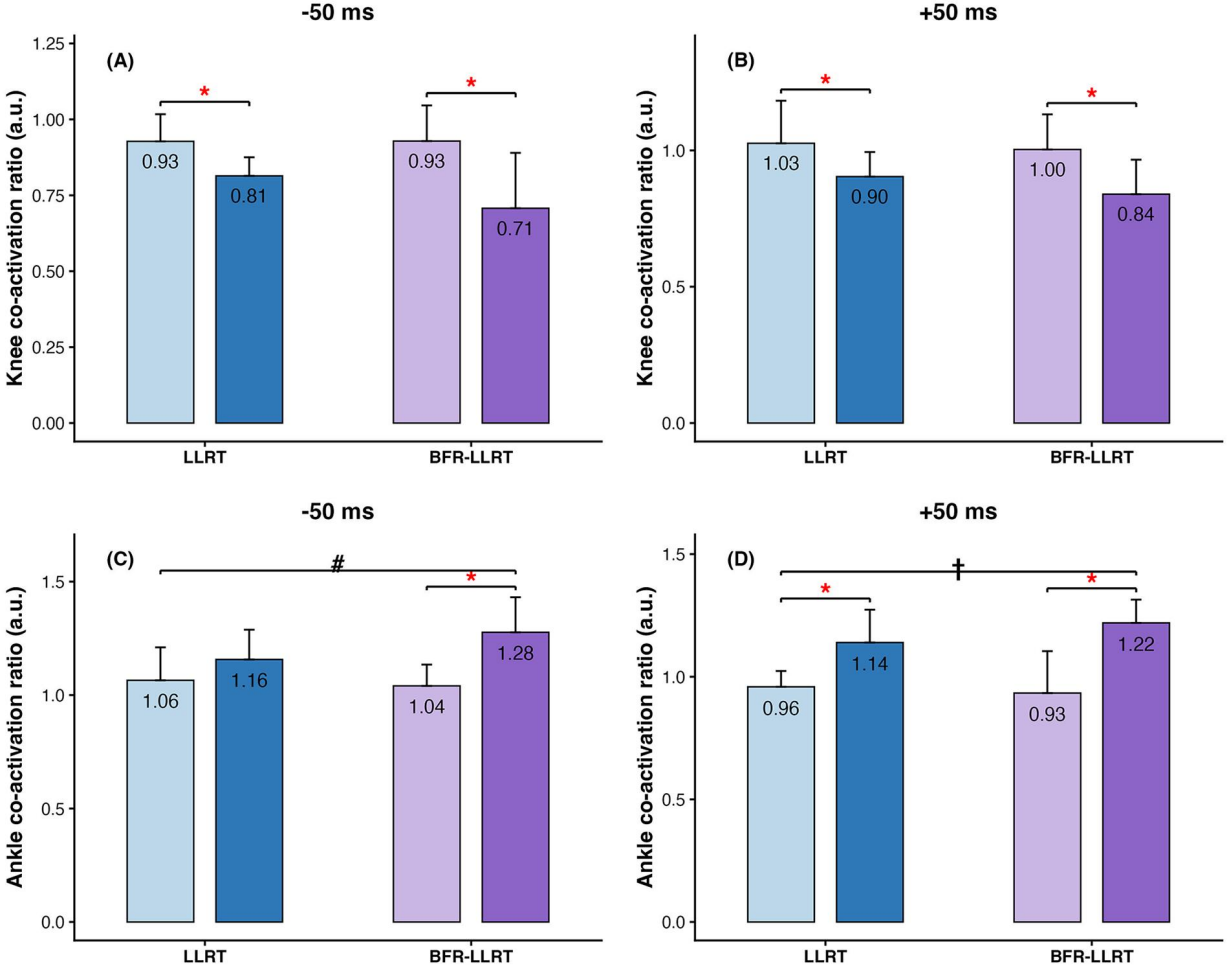
Changes in knee and ankle co-activation ratios at −50 ms and +50 ms relative to heel strike during obstacle crossing before and after the intervention. **(A,B)** show knee co-activation ratios, and **(C,D)** show ankle co-activation ratios at −50 ms and +50 ms, respectively. For each group, the left and right bars represent pre- and post-intervention values, respectively. Data are presented as mean ± SD. * *p* < 0.05 vs. pre-intervention within the same group; # *p* < 0.05 for the group × time interaction. Co-activation ratios are dimensionless.

Changes in neuromuscular activation are presented in Table 2 and Figure 1. Overall, changes in lower-limb co-activation patterns during obstacle crossing were observed after both LLRT and BFR-LLRT.

**Table 2:** Mixed ANOVA results for knee and ankle co-activation ratios.

Variable	Group F (*p*, *η*^2^)	Time F (*p*, *η*^2^)	Group × time F (*p*, *η*^2^)
Ankle co-activation ratio (−50 ms)	3.30 (0.077, 0.080)	24.65 (<0.001, 0.393)	4.75 (0.036, 0.111)
Ankle co-activation ratio (+50 ms)	0.93 (0.341, 0.024)	76.91 (<0.001, 0.669)	3.93 (0.055, 0.094)
Knee co-activation ratio (−50 ms)	6.36 (0.016, 0.143)	27.09 (<0.001, 0.416)	2.80 (0.103, 0.069)
Knee co-activation ratio (+50 ms)	2.27 (0.140, 0.056)	25.78 (<0.001, 0.404)	0.56 (0.460, 0.014)

Co-activation ratios were calculated as the ratio of antagonist to agonist muscle activation around the joint. Values at −50 ms and +50 ms represent mean activation within 50 ms before and after heel strike, respectively. EMG signals were normalized to peak activation across trials. Co-activation ratios are dimensionless (arbitrary units). *η*^2^ = partial eta squared.

For knee co-activation, a significant main effect of time was observed (*p* < 0.05), indicating reduced co-activation after training in both groups, whereas the group × time interaction was not significant (Table 2).

In contrast, a significant group × time interaction was found for ankle co-activation prior to heel strike (*p* < 0.05), reflecting a greater increase in the BFR-LLRT group (Table 2, Figure 1). No significant interaction was observed for ankle co-activation after heel strike.

Post hoc comparisons confirmed that both groups showed reductions in knee co-activation, whereas only the BFR-LLRT group exhibited a significant increase in ankle co-activation before heel strike (Table 2).

### 3.3 Obstacle-crossing performance

**Figure 2:**
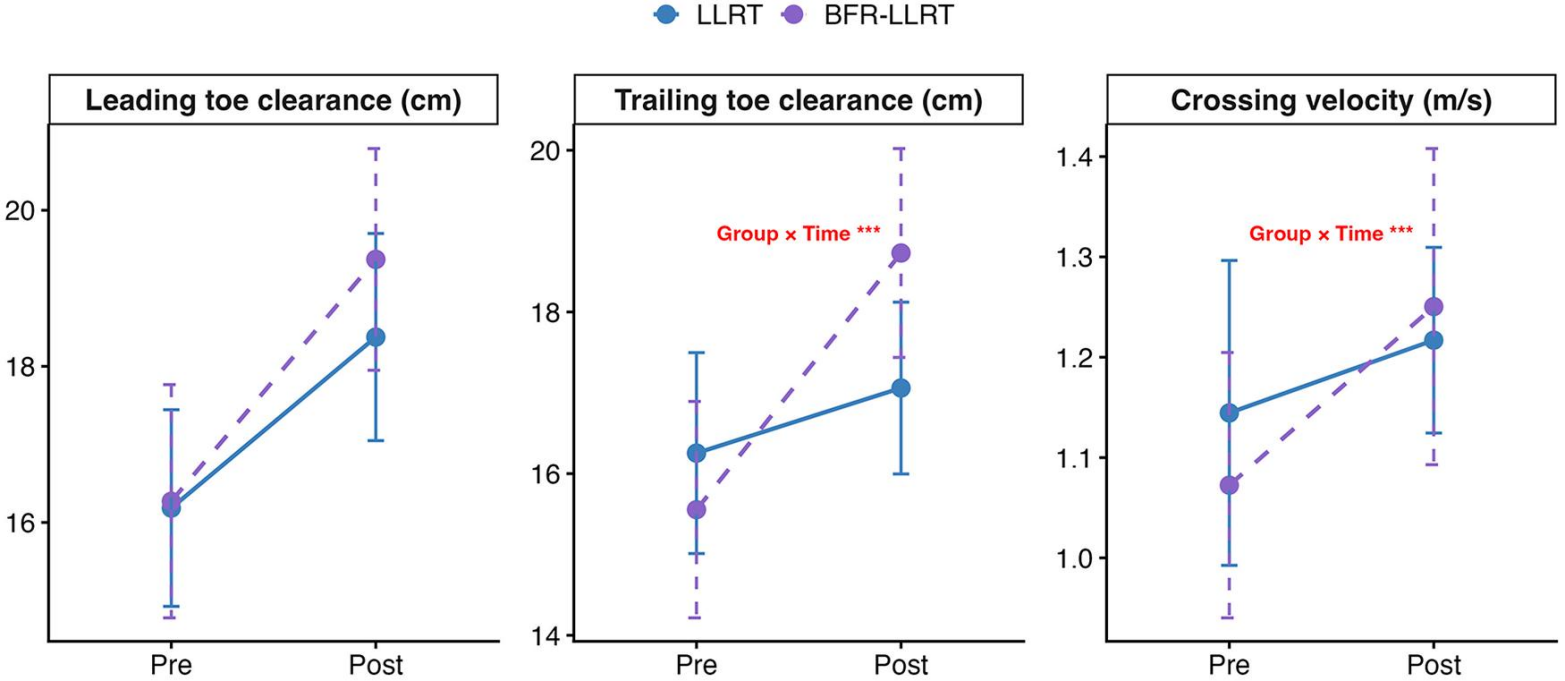
Changes in obstacle-crossing performance before and after the intervention. Panels represent leading-limb toe clearance, trailing-limb toe clearance, and crossing velocity. Data are presented as mean ± SD for the LLRT and BFR-LLRT groups. Significant group × time interactions were observed for trailing-limb toe clearance and crossing velocity, whereas the group × time interaction for leading-limb toe clearance did not reach statistical significance (*p* = 0.074). Units are indicated in each panel. *** *p* < 0.001.

Changes in obstacle-crossing performance are presented in Figure 2 and Table 3. For the primary outcome, trailing-limb toe clearance, a significant group × time interaction was observed, indicating a greater increase in the BFR-LLRT group. For the secondary obstacle-crossing outcomes, both groups showed increases in leading-limb toe clearance and crossing velocity; the group × time interaction was significant for crossing velocity but not for leading-limb toe clearance.

**Table 3:** Obstacle-crossing performance before and after the intervention.

Variable	LLRT pre	LLRT post	BFR-LLRT pre	BFR-LLRT post	Group × time (*p*, *η*^2^)
Leading toe clearance (cm)	16.18 ± 1.26	18.38 ± 1.33	16.27 ± 1.49	19.37 ± 1.42	*p* = 0.074, *η*^2^ = 0.081
Trailing toe clearance (cm)	16.25 ± 1.24	17.06 ± 1.06	15.55 ± 1.34	18.73 ± 1.29	*p* < 0.001, *η*^2^ = 0.320
Crossing velocity (m/s)	1.14 ± 0.15	1.22 ± 0.09	1.07 ± 0.13	1.25 ± 0.16	*p* < 0.001, *η*^2^ = 0.523

Data are presented as mean ± SD. *η*^2^ = partial eta squared.

Leading-limb toe clearance increased significantly over time in both groups, whereas the group × time interaction did not reach statistical significance (*p* = 0.074; Table 3).

Crossing velocity showed a significant group × time interaction (*p* < 0.001, partial *η*^2^ = 0.523), indicating greater increases in the BFR-LLRT group (Table 3).

Post hoc comparisons confirmed that both groups showed significant pre–post increases across all obstacle-crossing variables, whereas between-group differences at post-intervention were only observed for trailing-limb toe clearance (Table 3).

### 3.4 Balance performance

**Figure 3:**
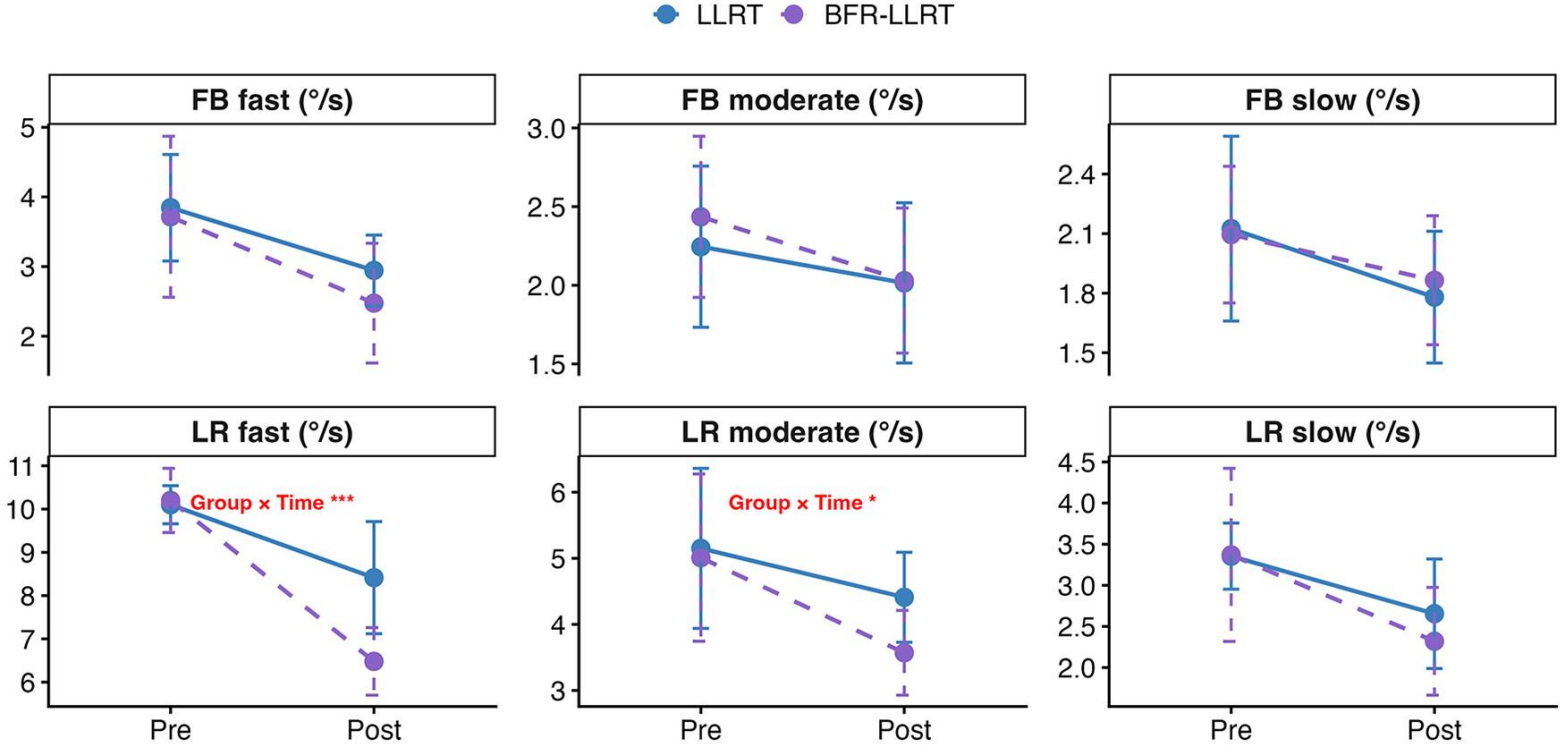
Changes in rhythmic weight-shift performance before and after the intervention. Panels represent anteroposterior and mediolateral directions at different movement speeds. Data are presented as mean ± SD for the LLRT and BFR-LLRT groups. Units are indicated in each panel. Red labels indicate conditions in which significant group × time interactions were observed. * *p* < 0.05; *** *p* < 0.001.

Changes in balance performance are summarized in Table 4 and Figure 3, with descriptive values for weight-bearing symmetry presented in Table 5. Overall, BFR-LLRT was associated with greater improvements in selected mediolateral dynamic balance outcomes, whereas effects on weight-bearing symmetry were angle-dependent.

**Table 4:** Mixed ANOVA results for balance outcomes.

Variable	Group F (p, *η*^2^)	Time F (p, *η*^2^)	Group × time F (*p*, *η*^2^)
RWS—Anteroposterior (fast)	1.53 (0.223, 0.039)	80.39 (<0.001, 0.679)	2.03 (0.162, 0.051)
RWS—Anteroposterior (moderate)	0.57 (0.455, 0.015)	15.49 (<0.001, 0.290)	1.18 (0.285, 0.030)
RWS—Anteroposterior (slow)	0.07 (0.793, 0.002)	27.97 (<0.001, 0.424)	1.12 (0.297, 0.029)
RWS—Mediolateral (fast)	22.24 (<0.001, 0.369)	192.54 (<0.001, 0.835)	27.29 (<0.001, 0.418)
RWS—Mediolateral (moderate)	3.45 (0.071, 0.083)	40.88 (<0.001, 0.518)	4.21 (0.047, 0.100)
RWS—Mediolateral (slow)	0.67 (0.417, 0.017)	49.17 (<0.001, 0.564)	1.97 (0.169, 0.049)
WBS symmetry (0°)	0.42 (0.519, 0.011)	9.59 (0.004, 0.202)	0.03 (0.868, <0.001)
WBS symmetry (30°)	10.08 (0.003, 0.210)	8.40 (0.006, 0.181)	18.34 (<0.001, 0.326)
WBS symmetry (60°)	11.27 (0.002, 0.229)	3.99 (0.053, 0.095)	11.17 (0.002, 0.227)
WBS symmetry (90°)	0.73 (0.397, 0.019)	37.15 (<0.001, 0.494)	12.49 (0.001, 0.247)

RWS, rhythmic weight shift; WBS, weight-bearing squat.

Anteroposterior and mediolateral denote movement directions in the RWS test.

Fast, moderate, and slow represent movement speed conditions.

WBS symmetry values indicate between-limb loading symmetry at different knee flexion angles (0°, 30°, 60°, and 90°).

Lower values indicate better between-limb symmetry.

*η*^2^ = partial eta squared.

**Table 5:** Weight-bearing symmetry before and after the intervention.

Variable	LLRT pre	LLRT post	BFR-LLRT pre	BFR-LLRT post	Group × time (*p*, *η*^2^)
WBS symmetry (0°)	7.50 ± 3.12	11.90 ± 7.64	8.60 ± 5.76	12.55 ± 6.61	*p* = 0.868, *η*^2^ < 0.001
WBS symmetry (30°)	8.55 ± 4.68	5.65 ± 3.62	6.50 ± 5.19	21.55 ± 17.43	*p* < 0.001, *η*^2^ = 0.326
WBS symmetry (60°)	14.50 ± 6.43	11.30 ± 5.64	13.25 ± 6.25	25.95 ± 16.54	*p* = 0.002, *η*^2^ = 0.227
WBS symmetry (90°)	23.05 ± 9.70	19.50 ± 5.92	26.20 ± 6.35	12.85 ± 8.61	*p* = 0.001, *η*^2^ = 0.247

Data are presented as mean ± SD. Lower values indicate better between-limb symmetry. *η*^2^ = partial eta squared.

For the Rhythmic Weight Shift (RWS) test, significant group × time interactions were observed in the mediolateral fast (*p* < 0.001, partial *η*^2^ = 0.418) and mediolateral moderate (*p* = 0.047, partial *η*^2^ = 0.100) conditions, indicating greater increases in on-axis velocity in the BFR-LLRT group (Table 4, Figure 3). No significant interactions were observed in the remaining conditions.

For weight-bearing symmetry, significant group × time interactions were found at 30° (*p* < 0.001), 60° (*p* = 0.002), and 90° (*p* = 0.001), whereas no interaction was observed at 0° (Table 4).

Post hoc comparisons indicated an angle-dependent pattern, with increased asymmetry at 30° and 60° but improved symmetry at 90° in the BFR-LLRT group (Table 5).

## 4 Discussion

The main findings indicate that BFR-LLRT was associated with task-specific changes in selected laboratory-based outcomes, including (1) changes in EMG-derived lower-limb co-activation indices around heel strike, particularly increased ankle co-activation before heel contact, (2) greater improvements in selected obstacle-crossing variables, especially trailing-limb toe clearance and crossing velocity, and (3) selective improvements in mediolateral dynamic balance. Overall, the original hypothesis was partially supported. BFR-LLRT was associated with greater changes in the primary outcome of trailing-limb toe clearance, crossing velocity, ankle co-activation before heel strike, and selected mediolateral dynamic balance outcomes. However, the hypothesis was not fully supported because favorable changes were not observed uniformly across all secondary outcomes, particularly Weight-Bearing Squat symmetry. Because muscle strength, joint kinetics, muscle morphology, and sensorimotor function were not directly assessed, these findings should be interpreted as associations among EMG-derived co-activation, kinematic performance, and balance outcomes rather than as direct evidence of a redistribution of neuromuscular control or a specific causal mechanism.

### 4.1 Neuromuscular activation

Both LLRT and BFR-LLRT were associated with changes in EMG-derived knee and ankle co-activation ratios around heel strike during obstacle crossing. The clearest between-group difference was observed for ankle co-activation prior to heel contact, whereas knee co-activation decreased over time in both groups without a significant interaction. These findings suggest that BFR-LLRT may be associated with greater ankle co-activation before heel strike during obstacle crossing.

The reduction in knee co-activation observed in both groups indicates a lower antagonist-to-agonist activation ratio around the knee after training. Although this pattern may be consistent with improved movement efficiency, this interpretation remains tentative because metabolic cost, movement efficiency, and the individual agonist and antagonist EMG components were not directly assessed or analyzed separately. Resistance training has been shown to decrease antagonist co-activation in older adults and may contribute to more efficient movement and improved motor control (17, 51). In the present study, the reduction in knee co-activation may therefore indicate a change in knee-level co-activation during obstacle crossing, but its functional meaning should be interpreted cautiously.

In contrast, the increase in ankle co-activation, particularly in the BFR-LLRT group, may be functionally relevant to ankle-level demands during obstacle crossing, although joint stiffness and kinetics were not directly measured. Previous experimental studies have shown that older adults increase ankle co-contraction under conditions requiring greater balance control, such as uneven or perturbed walking (52, 53). Although increased co-activation may reduce movement efficiency (54), in the context of obstacle crossing it may reflect a task-specific adjustment related to stability demands rather than a uniform improvement in neuromuscular efficiency.

Notably, the opposite directions of change observed in knee and ankle co-activation suggest joint-specific changes in EMG-derived co-activation patterns during obstacle crossing. Given that co-activation ratios are commonly used to describe antagonist–agonist activation around a joint during gait and balance-related tasks (30, 31, 41), these findings should be interpreted as joint-specific changes in EMG-derived co-activation indices. However, because the present study did not report the individual agonist and antagonist EMG components separately, and did not assess muscle strength, joint moments, muscle hypertrophy, or sensorimotor function, these EMG ratio findings cannot identify the specific physiological or biomechanical mechanisms underlying the observed changes. Therefore, the increased ankle co-activation before heel strike should be interpreted cautiously as a change in an EMG-derived co-activation index that may be related to ankle-level demands during obstacle negotiation.

### 4.2 Obstacle-crossing performance

Both LLRT and BFR-LLRT were associated with improvements in selected obstacle-crossing outcomes, as reflected by increases in toe clearance and crossing velocity. More consistent between-group differences were observed for trailing-limb toe clearance and the magnitude of improvement in crossing velocity, whereas leading-limb toe clearance improved in both groups and the group × time interaction did not reach statistical significance (*p* = 0.074). This pattern suggests that BFR-LLRT may be associated with greater changes in selected, more demanding components of obstacle negotiation (6, 13).

The increase in toe clearance may be biomechanically relevant to obstacle negotiation because insufficient toe clearance is a well-established contributor to obstacle contact and tripping risk in older adults (5, 50). However, increased toe clearance should not be interpreted exclusively as improved performance or safety. Older adults may also increase foot clearance as a compensatory or cautious locomotor strategy to reduce the likelihood of obstacle contact under challenging conditions. Therefore, the observed increase in toe clearance may reflect both changes in laboratory-based obstacle-crossing performance and a more conservative obstacle-negotiation strategy. Previous work has also shown that older adults adopt strategic kinematic adjustments during obstacle crossing, including increased toe-obstacle clearance, in response to more demanding conditions (55). In this context, the present findings suggest that both training interventions were associated with changes in selected laboratory-based obstacle-crossing outcomes and task-relevant kinematic variables.

In the present study, the increase in leading-limb toe clearance may reflect greater foot elevation during the initial crossing phase. However, the more pronounced improvement in trailing-limb toe clearance following BFR-LLRT is particularly noteworthy, because the trailing limb represents a more demanding phase of the task and requires continued limb elevation after the body has already progressed beyond the obstacle (6). Interventions targeting lower-limb function may therefore be relevant to obstacle negotiation, although the present study did not directly assess sensorimotor control or real-world trip risk.

Crossing velocity also increased in both groups, with a greater magnitude of improvement in the BFR-LLRT group. Because post-intervention values did not differ significantly between groups, this finding should be interpreted as a greater training-induced change rather than a higher final level of performance. Nevertheless, the ability to maintain forward progression during obstacle crossing is functionally important, as older adults often adopt slower and more conservative strategies under challenging conditions (5, 13). The greater increase in crossing velocity may therefore reflect a favorable training-related change in laboratory-based obstacle-crossing performance.

These kinematic improvements occurred concurrently with changes in EMG-derived co-activation patterns observed in the present study. Reduced knee co-activation and increased ankle co-activation occurred concurrently with changes in obstacle-crossing kinematics, but the present data cannot determine whether these EMG-derived changes contributed causally to improved performance. Taken together, these findings show that BFR-LLRT was associated with greater improvements in selected obstacle-crossing outcomes and with changes in EMG-derived co-activation patterns. Although these changes may be functionally relevant to obstacle negotiation, the present data do not establish that the improvements were mediated by coordinated neuromuscular or biomechanical adaptations. Future studies incorporating muscle strength testing, joint kinetics, muscle morphology, sensorimotor assessments, separate agonist and antagonist EMG analyses, and mediation models are needed to clarify the mechanisms underlying these performance changes.

### 4.3 Balance performance

The present study showed that BFR-LLRT was associated with improvements in selected balance-related outcomes, particularly mediolateral dynamic weight-shift performance, whereas changes in weight-bearing symmetry were angle-dependent and not uniformly favorable. This pattern suggests that the balance-related effects of BFR-LLRT were task-specific rather than generalized.

In the Rhythmic Weight Shift test, greater improvements were observed in mediolateral weight shifting at moderate and fast speeds following BFR-LLRT. Effective mediolateral control of the center of mass is important for maintaining stability during dynamic tasks, and impairments in this domain have been associated with fall-related risk factors in older adults (53, 56, 57). The present findings therefore suggest that BFR-LLRT may be associated with improvements in selected components of mediolateral dynamic balance.

These improvements may be related to training-associated changes in lower-limb function; however, the present study did not directly assess muscle strength, joint kinetics, muscle morphology, or sensorimotor function, and therefore cannot determine the mechanisms underlying these changes. Previous studies have reported that low-load blood flow restriction training may improve strength-related outcomes under low mechanical loads, which may be relevant to functional task performance (58). In the present study, improvements in selected mediolateral RWS outcomes may indicate favorable changes in laboratory-based dynamic weight-shift performance, particularly under conditions requiring rapid mediolateral weight transfer.

In contrast, the Weight-Bearing Squat results demonstrated a clear angle-dependent pattern. Symmetry improved at 90° knee flexion in the BFR-LLRT group, whereas increased asymmetry was observed at 30° and 60°. At 0°, asymmetry increased over time in both groups, but no significant group × time interaction was detected. These findings suggest that changes in bilateral loading were influenced by task demands, knee joint position, and mechanical constraints, rather than reflecting a uniform improvement in balance. Previous studies have similarly shown that postural control and loading strategies in older adults are highly context-dependent and vary according to task difficulty and biomechanical requirements (57).

Moreover, squat-related postural control and feet-loading symmetry are sensitive to task constraints such as stance width and body configuration, and lower-limb kinetic and center-of-pressure asymmetries can occur even during double-leg bodyweight squats (59, 60). Muscle activation patterns during squatting also vary according to knee flexion angle and center-of-pressure position, suggesting that WBS outcomes at different knee flexion angles may reflect angle-specific neuromuscular and mechanical demands (61).

Taken together, these findings indicate that BFR-LLRT was not associated with uniformly favorable changes across all balance-related outcomes. Rather, it may be associated with improvements in selected mediolateral dynamic balance outcomes, whereas its effects on bilateral loading control appear to be joint-angle dependent and not consistently beneficial. This task-specific and angle-dependent response may help explain why improvements in obstacle-crossing performance were not fully paralleled by favorable changes in all balance measures, highlighting the importance of considering task specificity and joint-position dependence when evaluating functional training outcomes in older adults.

### 4.4 Clinical interpretation and real-world relevance

Although the present findings showed statistically significant improvements in the primary outcome of trailing-limb toe clearance and in selected secondary outcomes, their clinical significance should be interpreted cautiously. Toe clearance during obstacle crossing is biomechanically relevant to tripping avoidance, and insufficient foot–obstacle clearance has been associated with an increased risk of obstacle contact in older adults (5, 6, 13, 34, 35). However, the present study did not directly assess real-world trips, falls, prospective fall incidence, or patient-reported mobility outcomes. Therefore, the observed improvements should be interpreted as evidence of improved laboratory-based obstacle-crossing performance rather than direct evidence of reduced fall or tripping risk in daily life.

To our knowledge, no established minimal clinically important difference or minimal clinically meaningful difference has been defined for trailing-limb toe clearance during obstacle crossing in older adults. Consequently, although the magnitude of improvement observed in the BFR-LLRT group may be biomechanically relevant in the context of obstacle negotiation, it remains uncertain whether this change translates into a clinically meaningful reduction in everyday tripping or falling. Future studies should establish clinically interpretable thresholds for obstacle-crossing variables and examine whether changes in toe clearance predict real-world fall outcomes.

In addition, obstacle crossing in the laboratory was performed under controlled conditions, with a standardized obstacle height and self-selected walking speed. Everyday obstacle negotiation is more complex and may involve variable obstacle dimensions, environmental distractions, dual-task demands, fatigue, visual constraints, and uneven surfaces (4, 9, 13, 55). Therefore, the generalizability of the present laboratory-based findings to daily-life mobility should be considered limited. Future research should incorporate more ecologically valid assessments, such as dual-task obstacle crossing, irregular obstacles, wearable sensor monitoring, and prospective fall surveillance.

Moreover, although fall history in the past 12 months was recorded, prospective fall incidence and real-world trip events during or after the intervention were not assessed. The sample also consisted of relatively high-functioning community-dwelling older adults who were able to walk independently. Therefore, the applicability of the findings to older adults with recurrent falls, frailty, or clinically diagnosed balance impairment remains unclear.

## 5 Conclusion

Both LLRT and BFR-LLRT were associated with changes or improvements in selected neuromuscular, obstacle-crossing, and dynamic balance-related outcomes in older adults. Compared with LLRT alone, BFR-LLRT was associated with greater changes in selected outcomes, including the primary outcome of trailing-limb toe clearance, crossing velocity, ankle co-activation prior to heel strike, and selected mediolateral dynamic balance measures. However, the effects on secondary outcomes were variable, and weight-bearing symmetry showed angle-dependent responses that were not consistently beneficial, with increased asymmetry at 30° and 60° knee flexion in the BFR-LLRT group but improved symmetry at 90°. Therefore, these findings should not be interpreted as evidence that BFR-LLRT uniformly improves balance performance. Overall, BFR-LLRT may be associated with additional improvements in selected laboratory-based outcomes in community-dwelling older adults, particularly trailing-limb toe clearance, crossing velocity, ankle co-activation before heel strike, and mediolateral dynamic balance. However, because the intervention consisted primarily of isolated lower-limb strengthening exercises and the locomotor outcomes were assessed under controlled laboratory conditions, these findings should not be interpreted as direct evidence of improved real-world mobility or clinical benefit. Nevertheless, because key mechanistic variables were not measured and mediation analysis was not performed, the present findings should not be interpreted as evidence of a specific neuromuscular, biomechanical, hypertrophic, or sensorimotor mechanism. Similarly, because real-world trip events, prospective fall incidence, and minimal clinically meaningful differences were not assessed or established, these findings should not be interpreted as direct evidence of reduced fall risk. Future research should include clinically relevant outcomes, longer follow-up periods, and ecologically valid mobility assessments to determine whether these laboratory-based improvements translate into meaningful real-world benefits.

## 6 Limitations

Several limitations should be acknowledged. First, although the sample size was sufficient to detect moderate effects for the primary outcome, it remained relatively small and may limit generalizability. The sample size calculation was based on the primary outcome, trailing-limb toe clearance, and the study may not have been sufficiently powered to detect smaller effects in secondary outcomes such as EMG-derived co-activation and balance variables. Second, the 8-week intervention period may not have been long enough to induce consistent adaptations across all outcomes, particularly for balance variables. Third, although neuromuscular activation was assessed using surface electromyography, the underlying mechanisms were not directly examined. Muscle strength, joint kinetics, muscle hypertrophy, and sensorimotor function were not assessed; therefore, the mechanisms underlying the observed changes should be interpreted cautiously. In addition, co-activation ratios were analyzed as composite antagonist-to-agonist indices, but the individual agonist and antagonist EMG components were not reported separately. This limits interpretation of whether changes in the ratios reflected reduced antagonist activity, increased agonist activity, or concurrent changes in both components. Moreover, no mediation analysis was performed; therefore, causal pathways linking EMG-derived co-activation changes to obstacle-crossing improvements cannot be inferred. Although additional baseline characteristics were reported, habitual physical activity level, baseline habitual walking speed, formal cognitive function, formal sensory status, detailed medication type and dosage, and direct muscle strength were not systematically assessed. Therefore, residual baseline differences in these unmeasured factors cannot be completely excluded. Fourth, participants were relatively high-functioning community-dwelling older adults who were able to walk independently, which may limit applicability to frailer older adults or clinical populations with recurrent falls or diagnosed balance impairments. Finally, no follow-up assessment was conducted. Although fall history in the past 12 months was recorded, the study did not assess prospective fall incidence, real-world trip events, patient-reported mobility outcomes, or clinically meaningful thresholds for obstacle-crossing performance. Therefore, the present findings cannot determine whether improvements in laboratory-based obstacle-crossing performance translate into reduced fall or tripping risk in daily life. Future studies should incorporate longer interventions, follow-up assessments, clinically relevant outcomes, prospective fall monitoring, and more ecologically valid mobility assessments.

## Data Availability

The raw data supporting the conclusions of this article will be made available by the authors, without undue reservation.
