# Supplementary figures and images for: Effects of blood flow restriction combined with low-load resistance training on obstacle-crossing performance and lower limb function in older adults
Source: Front Sports Act Living. 2026 Jul 1;8:1858963. doi: 10.3389/fspor.2026.1858963 (PMC13368982; doi:10.3389/fspor.2026.1858963)

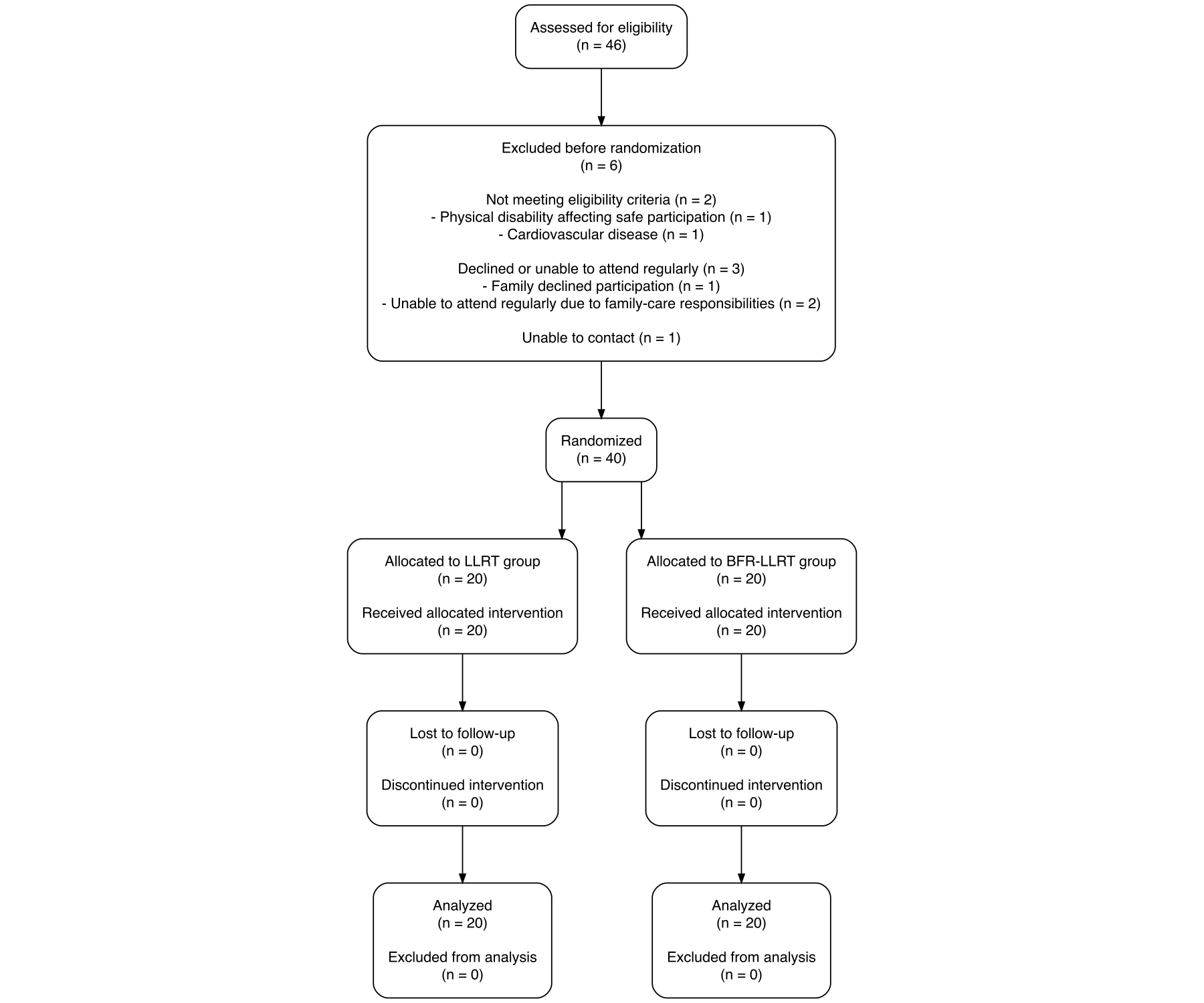

Supplement: Supplementary file 1 [file Image1.tiff]
