# Supplementary material for: Effects of blood flow restriction combined with low-load resistance training on obstacle-crossing performance and lower limb function in older adults
Source: Front Sports Act Living. 2026 Jul 1;8:1858963. doi: 10.3389/fspor.2026.1858963 (PMC13368982; doi:10.3389/fspor.2026.1858963)
Supplement: Supplementary file 2 [file Table1.docx]

**Supplementary Table 1. Training loads, BFR pressures, adherence, and safety outcomes during the intervention.**

| **Variable** | **LLRT group**  **(n = 20)** | **BFR-LLRT group**  **(n = 20)** |
| --- | --- | --- |
| Training frequency | 3 sessions/week | 3 sessions/week |
| Intervention duration | 8 weeks | 8 weeks |
| Target training intensity | Approximately 30%–40% estimated 1RM where external loading was applied | Approximately 30%–40% estimated 1RM where external loading was applied |
| Straight leg raise load (kg), range | 0.5–2 | 0.5–2 |
| Standing calf raise load | Body weight only | Body weight only |
| Seated knee extension load (kg), range | 8–13 | 8–13 |
| Sets × repetitions | 3 × 30 | 3 × 30 |
| Rest interval | 30–60 s | 30–60 s |
| Cuff size | — | B-STRONG size #4 |
| Cuff width × adjustable length (cm) | — | 7 × 54–79 |
| Recommended limb circumference range (cm) | — | 56.5–73.5 |
| Mean AOP (mmHg) | — | 185.9 ± 24.6 |
| Applied pressure at 50% AOP (mmHg) | — | 93.0 ± 12.3 |
| Applied pressure at 60% AOP (mmHg) | — | 111.5 ± 14.8 |
| Applied pressure at 70% AOP (mmHg) | — | 130.1 ± 17.2 |
| Pressure progression | — | Weeks 1–2: 50% AOP; weeks 3–5: 60% AOP; weeks 6–8: 70% AOP |
| Cuff release strategy | — | Released during each inter-set rest interval |
| Training adherence | ≥90% in all participants | ≥90% in all participants |
| Formal RPE scale recorded | No | No |
| Verbal monitoring of exertion/symptoms | Yes | Yes |
| Participants requiring modification/termination due to adverse symptoms, n | 0 | 0 |
| Participants with serious adverse events, n | 0 | 0 |
| Participants who discontinued the intervention, n | 0 | 0 |

Note. Data are presented as mean ± SD, range, or descriptive information as appropriate. Target training intensity refers to approximately 30%–40% of estimated 1RM for exercises with external loading. For standing calf raises, participants performed the exercise using body weight only; progression was controlled by movement quality, completion of repetitions, and tolerance rather than additional external loading. Applied cuff pressures were calculated as 50%, 60%, and 70% of each participant’s individualized AOP. Adverse symptoms refer to symptoms requiring exercise modification or termination. AOP = arterial occlusion pressure; BFR = blood flow restriction; BFR-LLRT = blood flow restriction combined with low-load resistance training; LLRT = low-load resistance training; RPE = rating of perceived exertion; 1RM = one-repetition maximum.
